# Application of pharmacologically induced transcriptomic profiles to interrogate PI3K-Akt-mTOR pathway activity associated with cancer patient prognosis

**DOI:** 10.18632/oncotarget.11776

**Published:** 2016-08-31

**Authors:** Matthew H. Ung, George L. Wang, Frederick S. Varn, Chao Cheng

**Affiliations:** ^1^ Department of Molecular and Systems Biology, Geisel School of Medicine at Dartmouth, Hanover, New Hampshire, 03755 USA; ^2^ Department of Biomedical Data Science, Geisel School of Medicine at Dartmouth, Lebanon, New Hampshire, 03755 USA; ^3^ Norris Cotton Cancer Center, Geisel School of Medicine at Dartmouth, Lebanon, New Hampshire, 03766 USA

**Keywords:** pharmacogenomics, computational biology, PI3K, drug treatment

## Abstract

The PI3K-Akt-mTOR signaling pathway has been identified as a key driver of carcinogenesis in several cancer types. As such, a major area of focus in cancer biology is the development of genomic biomarkers that can measure the activity level of the PI3K-Akt-mTOR pathway. In this study, we systematically estimate PI3K-Akt-mTOR pathway activity in breast primary tumor samples using transcriptomic profiles derived from drug treatment in MCF7 cell lines. We demonstrate that gene expression profiles derived from chemically-induced protein inhibition allows us to measure PI3K-Akt-mTOR pathway activity in patient tumor samples. With this approach, we predict prognosis and response to chemotherapy in cancer patients, and screen for potential pharmacological modulators of PI3K-Akt-mTOR pathway inhibitors.

## INTRODUCTION

In cancer, genomic lesions lead to constitutive activation of signaling pathways that induce uncontrolled cellular proliferation and confer a survival advantage upon transformed cells. Thus, being able to accurately identify the key molecular drivers underlying each patient's tumor would significantly accelerate the development of precision medicine. Fortunately, studies have already identified several signaling pathways that are recurrently dysregulated, one of which is the PI3K-Akt-mTOR pathway. The PI3K-Akt-mTOR pathway is composed of a number of proteins including Ras, PTEN, PIP, PI3K, AKT and mTOR. The pathway functions as part of a signaling cascade initiated by the binding of growth factors to receptor tyrosine kinases located on the cell membrane [1, 2]. Importantly, the PI3K-Akt-mTOR pathway plays a central role in cancer cell growth, proliferation, and survival, and is overexpressed in numerous cancers including breast, ovarian, and pancreatic [3–5]. Consequently, there have been significant efforts in developing PI3K inhibitors that can abrogate tumor growth and act synergistically with other targeted therapy and chemotherapy [1, 6, 7].

Due to the importance of this pathway, several genomics-based approaches have been introduced to assess the activity of the PI3K-Akt-mTOR pathway in cell lines and in primary patient tumors. A common method is to determine whether a gain-of-function mutation in *PIK3CA*, which encodes the PI3K catalytic subunit, p110α is present in patient tumors. However, conflicting results have risen from these studies, with some suggesting that *PIK3CA* mutations are associated with poor prognosis, and others suggesting the opposite [8–11]. Reasons for this inconsistency include varying effects of *PIK3CA* mutation in different cancer types and the fact that *PIK3CA* mutations can co-occur with molecular events that modulate *PIK3CA* mutational effects. Other approaches to assessing PI3K-Akt-mTOR pathway activity include using signature-based approaches that capture the gene expression change caused by *PIK3CA* mutations [12]. For instance, Loi et al. used gene expression profiles from primary breast tumors containing *PIK3CA* mutations to design a signature of PI3K activation to predict PI3K-Akt-mTOR pathway activity in an independent dataset [12]. In general, they found that patients with breast tumors displaying a *PIK3CA* mutation-like expression pattern exhibited poor survival. However, results were inconsistent in ER+/HER2-breast tumors where they found that a *PIK3CA* mutant gene signature was associated with improved survival [12].

Despite the utility of such approaches, using mutation-based analyses is subject to confounding by co-occurring genomic lesions. Since mutations may not always occur independently from each other, using a signature that is associated with a single mutation (i.e. *PIK3CA*) may also capture the effects of co-occurring lesions that may yield unreliable results when the goal is to specifically measure PI3K activity [13]. Therefore, in this proof of concept study, we utilized the drug treatment profiles of three PI3K-Akt-mTOR pathway inhibitors—LY294002 (reversible PI3K inhibitor), wortmannin (non-reversible PI3K inhibitor), and sirolimus (mTOR inhibitor)—to develop expression-based markers of PI3K-Akt-mTOR pathway activity. Since these three drugs target the PI3K-Akt-mTOR pathway, we can directly analyze the downstream effects of protein inhibition that occur solely due to ablation of PI3K catalytic activity while maintaining its non-catalytic biological functions [14]. To our knowledge, there has been no other study that has used pharmacological protein inhibition to probe cancer driver pathways in primary tumors. Overall, our analysis framework presents a novel method of utilizing protein inhibition gene expression profiles achieved by treating cell lines with targeted chemical inhibitors.

## RESULTS

### Overview of systematic analysis framework

**Figure 1:**
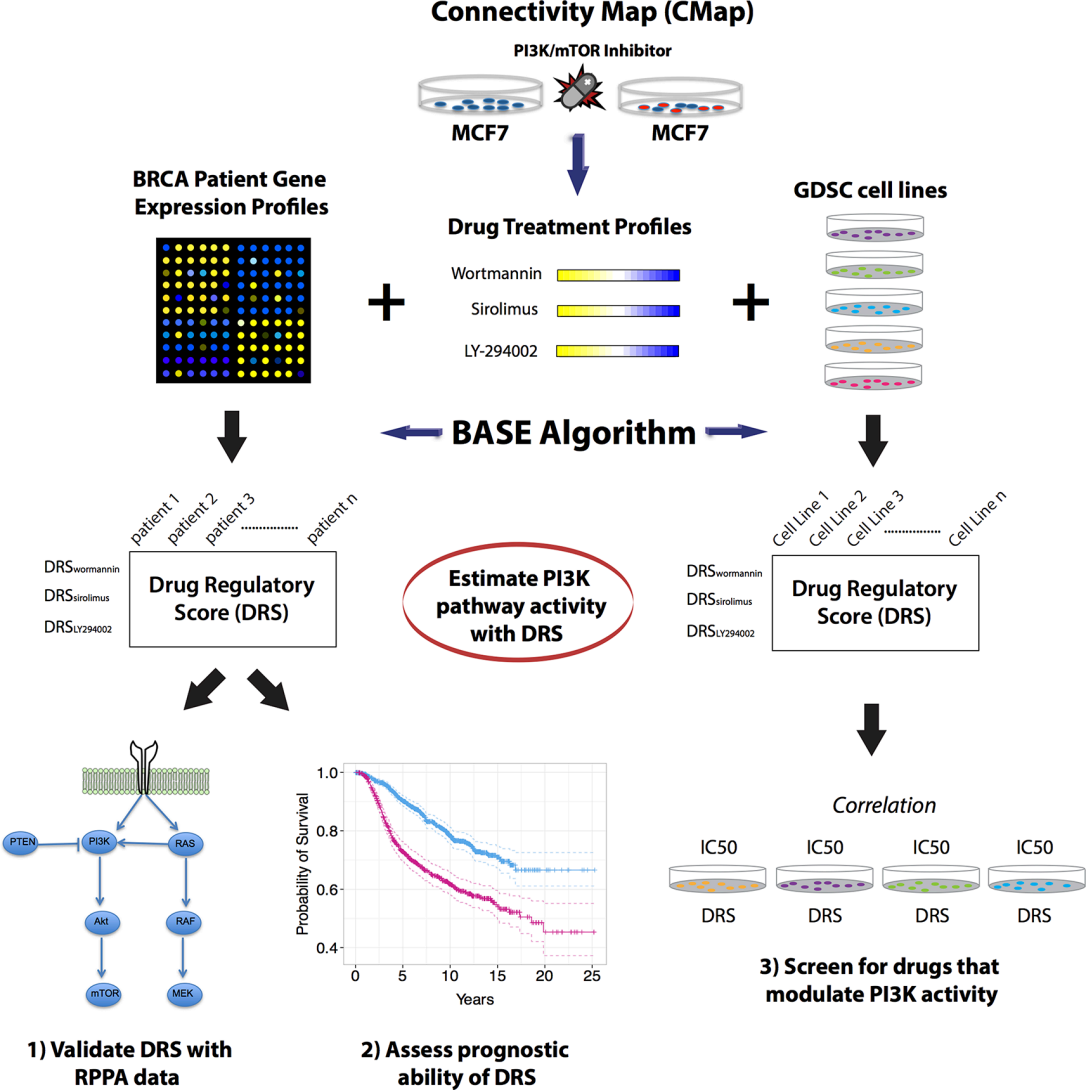
Overview of the analysis approach (**A**) Drug treatment profiles were generated from MCF7 cell lines that were treated with three different PI3K-Akt-mTOR pathway inhibitors (LY294002, wortmannin, sirolimus). Drug treatment profiles were used to calculate a DRS for patient tumors in several breast cancer datasets and for GDSC cell lines. DRS was validated in the TCGA dataset using RPPA data. After validation of DRS as estimator of PI3K-Akt-mTOR pathway activity, DRS was used to predict patient prognosis in breast cancer datasets. DRS was also calculated for GDSC cell lines and correlated with drug IC_50_ of 139 compounds.

First, in our systematic analysis, we generated drug treatment profiles corresponding to three PI3K inhibitors: LY294002 (reversible PI3K inhibitor), wortmannin (non-reversible PI3K inhibitor), and sirolimus (mTOR inhibitor) using data from the Connectivity Map (CMap) (Figure 1). Each drug treatment profile was generated by comparing the gene expression profile of MCF7 cells treated with one of these inhibitors to the gene expression profile of untreated MCF7 cells. As such, we derived a total of three drug treatment profiles that encode the change in gene expression induced by each of the three inhibitors.

Second, we utilized the BASE algorithm to compare each drug treatment profile to every patient cancer gene expression profile in The Cancer Genome Atlas (TCGA), METABRIC, van de Vijver, Loi, and Hatzis breast cancer datasets [15–19]. The algorithm outputs a drug regulatory score (DRS) which is a quantitative measure of similarity between each drug treatment profile and each patient gene expression profile. BASE uses entire gene expression profiles to calculate the DRS from continuous values, which obviates the need to set arbitrary cutoffs to define binary gene sets. As such, a DRS > 0 indicates that a tumor's gene expression pattern resembles the pattern that is induced by the drug. Conversely, a DRS < 0 indicates that a tumor exhibits a gene expression pattern opposite of what is induced by the drug. This is equivalent to the concept that a high tumor DRS will be assigned if genes upregulated by inhibitor treatment are also highly expressed in the tumor and genes downregulated by treatment are lowly expressed in the same tumor.

Moreover, we used these DRSs as estimators of PI3K-Akt-mTOR pathway activity in TCGA patient tumors and validated its accuracy using corresponding patient Reverse Phase Protein Array (RPPA) data. After validation, we performed survival analysis using patient clinical information to determine if DRS could predict patient prognosis and response to chemotherapy in histological and molecular subtypes of breast cancer.

Finally, we repeated the analysis in cell lines available from the Genomics of Drug Sensitivity (GDSC) database given that they contained corresponding gene expression profiles. By generating a DRS for each GDSC cell line, were able to correlate DRS with drug sensitivity to screen for potential therapeutic modulators of these PI3K inhibitors.

### PI3K-Akt-mTOR pathway inhibitor profiles reveal PI3K-Akt-mTOR pathway activity in breast cancer subtypes

**Figure 2:**
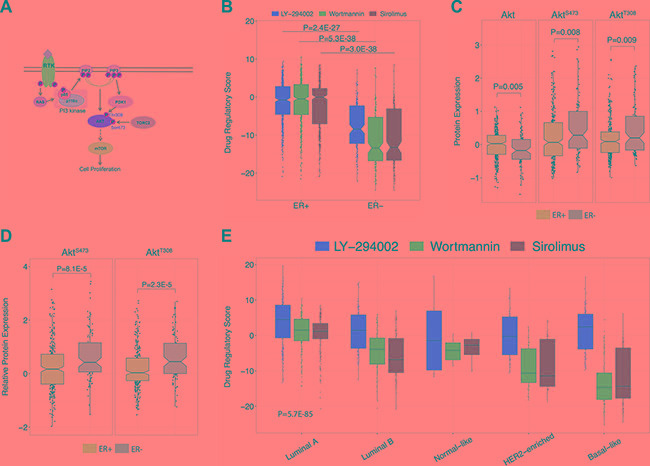
Comparison of DRS between molecular and histological breast tumor subtypes (**A**) Graphical overview of the PI3K-Akt-mTOR pathway. (**B**) Comparison of DRSs between ER+ and ER- breast tumors. Each point represents a single primary tumor sample. The black line within the boxes represent the median. The ER+ group had 598 samples and the ER- group had 178 samples. (**C**) Akt, pAkt^S473^, and pAkt^T308^ protein expression differences between ER+ and ER– breast tumors. Protein expression levels were derived from RPPA data. The ER+ group had 608 samples and the ER– group had 186 samples. (**D**) Differences in pAkt^S473^/Akt and pAkt^T308^/Akt expression ratios between ER+ and ER– breast tumors. The ER+ group had 608 samples and the ER– group had 186 samples. (**E**) Comparison of DRSs between Luminal A, Luminal B, Normal-like HER2, and Basal breast tumor subtypes. There were 231, 127, 97, 58, and 8 samples available for Luminal A, Luminal B, Basal-like, HER2-enriched, and Normal-like tumors, respectively. (The boxplot boundaries represent the 25th (lower black line), 50th (center black line), and 75th (upper black line) percentiles respectively. The whiskers are the upper and lower adjacent values).

The PI3K signaling pathway is strongly associated with the development and/or progression of several cancers. Thus, constitutive activation or ablation of proteins in the PI3K-Akt-mTOR pathway can result in uncontrolled cellular proliferation and tumor growth (Figure 2A). As such, we generated DRS profiles corresponding to each drug across patient tumors in the TCGA breast cancer dataset to determine if clinicopathological subgroups of patients differ in terms of their DRS. Since tumor subtyping is standard clinical protocol used to inform patients about prognosis and potential treatment options, we hypothesized that DRS could elucidate differences in PI3K-pathway activity in histological and molecular subtypes of breast cancer.

In particular, we stratified breast cancer patient tumors from TCGA on estrogen receptor (ER) status and compared them based on DRS_LY-294002_, DRS_wortmannin_, and DRS_sirolimus_. We found that ER+ tumors exhibited much higher DRS_LY-294002_ (*P* = 2.4E-27, Wilcoxon rank-sum test), DRS_wortmannin_ (*P* = 2.1E-49, Wilcoxon rank-sum test) and DRS_sirolimus_ (*P* = 2.9E-36, Wilcoxon rank-sum test) compared to ER- tumors (Figure 2B), indicating that ER+ tumors exhibit lower PI3K-pathway activity since it is more similar to a PI3K/mTOR-inhibited profile. This is consistent with previous studies demonstrating that PI3K-Akt-mTOR pathway activity is inversely correlated with ER expression, can function as a compensatory pathway that drives anti-estrogen resistance, and is required for hormone independence [20–22]. To validate the DRS results, we compared Akt, pAkt^S473^, and pAkt^T308^ protein expression levels between ER+ and ER- breast tumor samples using TCGA RPPA data. (Figure 2C) [18]. Unexpectedly, we observed increased expression of Akt in ER+ tumors suggesting enhanced PI3K-pathway activity. However, after closer analysis, we found that pAkt^S473^ and pAkt^T308^ expression was significantly decreased in ER+ tumors indicating that activated pAkt levels are lowered in ER+ tumors suggesting decreased PI3K-Akt-mTOR pathway activity (*P* = 0.008 and *P* = 0.009, respectively, Wilcoxon rank-sum test). However, higher pAkt levels only indicate that there is a higher amount of phosphorylated Akt in a tumor, and does not directly inform us of the overall proportion of activated pAkt compared to un-activated Akt. For instance, if a tumor typically has high basal expression of Akt, then it could also harbor higher quantities of pAkt. Thus, we calculated a ratio of phosphorylated pAkt to unphosphorylated Akt and compared the ratios between ER+ and ER- tumors. Tumors with a higher ratio would have a higher percentage of the activated (phosphorylated) Akt, thus indicating increased pathway activity. Confirmatively, we observed that ER+ tumors contain less activated pAkt relative to the pool of un-activated Akt for both pAkt^S473^ (*P* = 8.1E-5, Wilcoxon rank-sum test) and pAkt^T308^ (*P* = 2.3E-5, Wilcoxon rank-sum test) (Figure 2D), indicating that Akt RPPA data are consistent with DRS. We extended this analysis to other proteins downstream of Akt including GSK3, S6K1, and 4E-BP1 and observed consistent trends in protein expression (Supplementary Figure S1).

In addition to ER status, we investigated differences in DRSs between intrinsic subtypes of breast cancer. In particular, we observed that luminal A breast cancers had the highest DRSs while basal breast cancers had the lowest DRSs (*P* = 5.7E-85, ANOVA) (Figure 2E), indicating that luminal A and basal breast carcinomas exhibit the lowest and highest PI3K-Akt-mTOR pathway activity, respectively. Indeed, several studies have reported basal breast carcinomas to be an aggressive subtype that responds poorly to targeted therapy [23].

### DRS reveals confounding effect of PTEN expression on *PIK3CA* mutation and *PIK3CA* expression analysis

**Figure 3:**
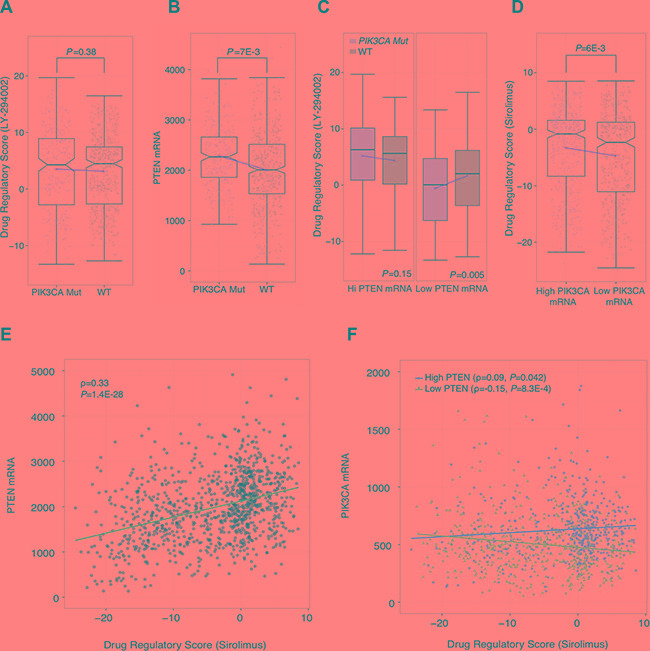
Stratified analysis using drug regulatory scores (**A**) Comparison of DRS_LY-294002_ between PIK3CA mutant and wild-type breast tumors. Each point represents a single tumor sample. Plot shows 507 wild-type samples and 258 PIK3CA mutant samples. (**B**) Differences in PTEN mRNA expression compared between PIK3CA mutant and wild-type breast tumors. Plot shows 507 wild-type samples and 258 PIK3CA mutant samples (**C**) Comparison of DRS_LY-294002_ between PIK3CA mutant and wildtype samples in high and low PTEN mRNA expression backgrounds (top 50% pTEN expression; left) and low PTEN mRNA expression (bottom 50% PTEN expression; right). There were 179, 340, 79 and 440 samples in the Hi PTEN/PIK3CA Mutant, Hi PTEN/WT, Low PTEN/PIK3CA Mutant, and Low PTEN/WT groups, respectively. (**D**) Differences in DRS_sirolimus_ between breast tumors with higher vs. lower PIK3CA mRNA expression. Each point represents a single tumor sample. The high PIK3CA mRNA group had 518 samples and the low PIK3CA mRNA group had 519 samples. (**E**) Correlation between breast tumor PTEN mRNA expression and DRS_sirolimus_ across 1037 samples. (**F**) Correlation between breast tumor PIK3CA mRNA expression and DRS_sirolimus_; segmented between high and low (top 50% and bottom 50%) PTEN mRNA expression level. High PTEN group had 514 samples, and the Low PTEN group had 517 samples. (The boxplot boundaries represent the 25th (lower black line), 50th (center black line), and 75th (upper black line) percentiles respectively. The whiskers are the upper and lower adjacent values. Red line indicates difference in mean between the two groups).

Since we used PI3K inhibitor profiles to delineate PI3K-Akt-mTOR pathway activity, we reasoned that our DRSs should be consistent with genetic and expression markers of PI3K-Akt-mTOR pathway activity. Several studies have reported *PIK3CA* mutation status to be associated with improved response to PI3K inhibitors [24, 25]. *PIK3CA* encodes p110α, a catalytic subunit of PI3K, and gain-of-function mutations in this gene have been reported to elevate signaling of cellular proliferation, growth, and metastasis [26–29]. To investigate the consistency of DRS with these observations, we first stratified TCGA breast cancer patient samples on *PIK3CA* mutation status and compared their DRS_LY-294002_. Surprisingly, we found no significant difference in DRS_LY-294002_ between *PIK3CA* mutant and wild-type (WT) tumor samples (Figure 3A). Since PI3K-Akt-mTOR pathway activity is not solely determined by PI3K alone, we postulated that genetic alteration of *PIK3CA* may be correlated with another alteration to an inhibitory protein of PI3K. Therefore, we investigated the most likely candidate, PTEN, and its expression in *PIK3CA* mutant and WT samples We observed that PTEN expression was significantly upregulated in *PIK3CA* mutant samples (*P* = 7E-3, Wilcoxon rank-sum test) (Figure 3B). This lead us to suspect that *PIK3CA* mutations are intertwined with PTEN transcriptional activity, which may indicate that 1) upregulation of PTEN expression was a cellular response to earlier cancer-driving events prior to *PIK3CA* mutation, and positive selection for *PIK3CA* mutations enabled tumors to ameliorate the suppressive effects of PTEN upregulation, or 2) *PIK3CA* mutations initially resulted in an early survival advantage but was eventually suppressed by upregulated PTEN expression as part of a protective cellular response. Moreover, either of these events are possible depending on the individual tumor's evolutionary history.

To evaluate this postulation, we performed a stratified analysis by categorizing patient tumors into high and low PTEN expression groups using median PTEN expression as the cutoff. We then compared DRS_LY-294002_ between *PIK3CA* mutant and wild-type samples in both the high and low PTEN groups. We observed that in the high PTEN expression group, there were no significant differences in DRS_LY-294002_. However, we found that in the low PTEN expressing group, *PIK3CA* mutant samples had significantly lower DRS_LY-294002_ compared to wild-type samples indicating that they had higher PI3K-Akt-mTOR pathway activity (*P* = 5E-3, Wilcoxon rank-sum test) (Figure 3C). These results suggest that *PIK3CA* mutants do confer increased pathway activity but only in the background of low PTEN expression. This demonstrates that DRS, in general, is an indicator of overall pathway activity, and not just PI3K activity. Furthermore, these results imply that *PIK3CA* mutation status may not be the most accurate indicator of PI3K-Akt-mTOR pathway activity due to confounding effects by PTEN.

Furthermore, we evaluated concordance of DRS_sirolimus_ with *PIK3CA* gene expression by stratifying TCGA patient samples into high and low *PIK3CA* gene expression groups using median expression as the cutoff. Similar to the mutation analysis, we found that DRS_sirolimus_ was significantly higher in tumors with high *PIK3CA* expression, which initially seemed to suggest that high *PIK3CA* expression is correlated with high DRS and decreased PI3K-Akt-mTOR pathway activity (*P* = 6E-3, Wilcoxon rank-sum test) (Figure 3D). However, we found that PTEN expression was also significantly correlated with DRS_sirolimus_ (PCC = 0.33, *P* = 1.4E-28) (Figure 3E). Therefore, we stratified patient samples into high and low PTEN expression groups and correlated *PIK3CA* expression with DRS in both strata. Our analysis shows that in low PTEN expression tumors, *PIK3CA* expression is anti-correlated with DRS_sirolimus_, indicating that increased *PIK3CA* mRNA is associated with decreased DRS_sirolimus_ and thus higher PI3K-Akt-mTOR pathway activity (PCC = –0.15, *P* = 8.3E-4) (Figure 3F). Moreover, we observed *PIK3CA* expression to be correlated with DRS_sirolimus_ in high PTEN expression tumors (PCC = 0.09, *P* = 0.04).

Together, these results suggest that *PIK3CA* mutation and expression is associated with PTEN expression, which again may be a reflection of the competition that occurs between pro-cancer molecular programs and anti-cancer cellular responses. Since DRS captures the entire expression output that results from the inhibition of a key PI3K-Akt-mTOR pathway regulator, it is more representative of overall pathway activity than *PIK3CA* mutation or *PIK3CA* expression alone, which may be confounded by the activity of other proteins in the pathway.

### PI3K-Akt-mTOR pathway inhibitor profiles predict prognosis for breast cancer patients

Since over-activation of the PI3K-Akt-mTOR pathway has been reported to be associated with cellular proliferation and patient prognosis, we investigated the clinical implications of high and low DRS in breast cancer patients. First, we investigated if DRSs were associated with proliferation—a key indicator of patient survival—and found that DRS_wortmannin_ was significantly anti-correlated with Ki67 mRNA expression in the METABRIC dataset (PCC = –0.62, *P* = 1.4E-53). Ki67 is a well-established cellular marker of proliferation and its correlation with DRS_wortmannin_ shows that DRS captures information about the proliferative state of the patient tumor [30, 31]. Furthermore, this suggests that DRS is able to capture the downstream proliferative signals associated with varying levels of PI3K-Akt-mTOR pathway activity.

**Figure 4:**
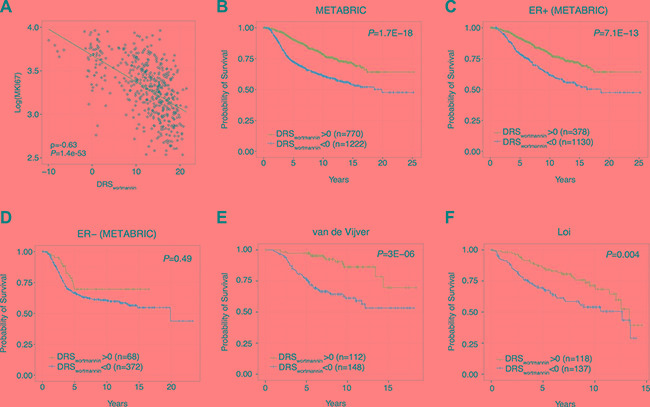
Survival analysis in breast cancer using DRS (**A**) Correlation between breast tumor DRS_wortmannin_ and Ki67 expression. Each dot point represents a single tumor sample. (**B**) Kaplan-Meier survival curves of breast cancer patients with DRS_wortmannin_ greater than or less than 0. Each vertical line represents a censored patient. (**C**–**D**) Kaplan-Meier survival curves of ER+ and ER- breast cancer patients with DRS_wortmannin_ greater and less than 0. (**E**–**F**) Kaplan-Meier survival curves of breast cancer patients using two additional datasets from van de Vijver et al. and Loi et al. [32, 34].

Second, we aimed to confirm the prognostic significance of DRS by analyzing its association with patient survival. After calculating a DRS_wortmannin_ for each sample, we stratified tumors into a high DRS_wortmannin_ (> 0) and a low DRS_wortmannin_ (< 0) group and compared their survival rates. In the METABRIC dataset, we found that patients with DRS_wortmannin_ > 0 exhibited significantly improved survival compared to patients with DRS_wortmannin_ < 0 (*P* = 1.7E-18, Log-rank test) (Figure 4B). This indicates that patient tumors with basal expression similar to the gene expression profile that is induced by wortmannin have better prognosis. Second, we aimed to investigate if the observed differences in survival between high and low DRS_wortmannin_ patients vary according to ER status. Interestingly, our analysis shows that DRS_wortmannin_ predicts prognosis in ER+ samples (*P* = 7.1E-13, Log-rank test) but not in ER- samples (*P* = 0.49, Log-rank test), which may suggest that there are interactions between ERα and PI3K activity that affect patient survival (Figure 4C, 4D). To test reproducibility, we analyzed additional breast cancer datasets published by van de Vijver et al. and Loi et al. [12, 19]. In the van de Vijver dataset, we found that patients with high DRS_wortmannin_ presented improved survival compared to low DRS_wortmannin_ patients (*P* = 3E 6, Log-rank test) [19]. Furthermore, we observed similar results in the Loi dataset with high DRS_wortmannin_ patients exhibiting favorable prognosis (*P* = 4E-3, Log-rank test) [17]. Together, our results demonstrate that calculation of DRS_wortmannin_ in patient tumors allows us to distinguish those patients with a more favorable prognosis.

Third, we investigated whether the cell line from which our drug treatment profiles were derived could impact the results of our survival analyses. Since CMap contains gene expression profiles for PC3 (prostate cancer) and HL60 (promyelocytic leukemia) cell lines, we postulated that DRS derived from these drug treatment profiles would be less prognostically significant when applied to breast cancer [32]. As expected, we found that DRS calculated from MCF7-derived drug treatment profiles were the most prognostic in the METABRIC dataset (Supplementary Table S1). Since MCF7 is a breast-derived cell line, our results support our claim that using drug treatment profiles from matched cell lines yields optimal results when evaluating patient survival.

### PI3K-Akt-mTOR pathway inhibitor profiles predict response to taxane therapy in breast cancer

Although we confirmed that DRS was highly prognostic in primary patient tumors, we acknowledge that these gene expression profiles were collected from patients who have not yet received chemotherapy. Thus, drug treatment may substantially alter the basal expression of pre-treated tumors leading to a change in prognosis. To determine if DRS could also serve as a predictor of response to taxane-anthracycline chemotherapy, we utilized a dataset published by Hatzis et al. which contains gene expression profiles from pre-treated primary tumors and survival information of the patients as they receive neoadjuvant taxane-anthracycline chemotherapy [16]. Additionally, this dataset contains information about whether treated patients had high or low residual cancer burden.

**Figure 5:**
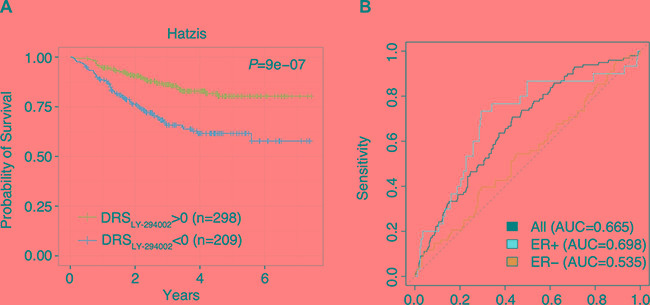
DRS predicts response to taxane-anthracycline chemotherapy (**A**) Kaplan-Meier survival curves of taxane-anthracycline chemotherapy-treated breast cancer patients with DRS_LY-294002_ greater and less than 0. Each vertical line represents a censored patient. (**B**) Receiver Operating Characteristic (ROC) curve for random forest classification predicting residual cancer burden using DRS_LY-294002_. Random forest classification was conducted using (1) all breast tumors, (2) ER+ breast tumors only, and (3) ER– breast tumors only.

First, we found that DRS_LY-294002_ was predictive of patient distant relapse free survival, indicating that DRS can serve as a predictive marker of patient response to chemotherapy (*P* = 9.0E-7, Log-rank test) (Figure 5A). Furthermore, we evaluated whether DRS_LY-294002_ could predict residual cancer burden, another key indicator of chemotherapy responsiveness in the Hatzis dataset [16]. In particular, we stratified patients into two groups, the first corresponding to low residual cancer burden and the second corresponding to high residual cancer burden. We then trained a random forest classifier on patient DRS_LY-294002_ and found that the model was able to predict residual cancer burden with an AUC of 0.665 (Figure 5B). Furthermore, we repeated the analysis in ER+ and ER- patient samples and found that classification accuracy was higher in ER+ (AUC = 0.698) than in ER- (AUC = 0.535) cancers. Since MCF7 is an ER+ cell line, these results suggest that using matched cell lines yields improved results. Together these results indicate that PI3K-Akt-mTOR pathway activity is a key predictor of response to neoadjuvant chemotherapy and can be interrogated using drug treatment profiles. Indeed, previous studies have demonstrated that increased PI3K-Akt-mTOR pathway activity is associated with chemoresistance in patients and in breast cancer cell lines [29, 33].

### Systematic screening of therapeutics associated with PI3K-Akt-mTOR pathway activity

**Figure 6:**
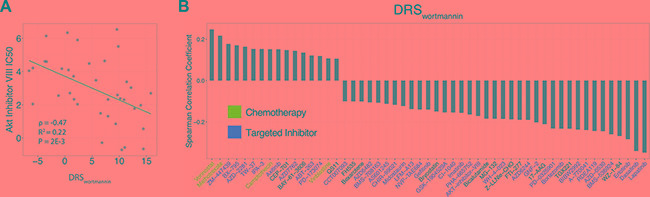
Pharmacological correlates of PI3K-Akt-mTOR pathway activity (**A**) Correlation of DRS with AKT Inhibitor VIII IC_50_. Each point represents a single cell line. (**B**) Correlation between DRS and the IC_50_ of 53 drugs. Blue colored drug names are chemotherapeutics and red colored drug names are targeted inhibitors.

Because DRS is calculated using the gene expression profile of a chemical inhibitor, we can also use DRS to investigate if other potential compounds can modulate that inhibitor's activity. Thus, we carried out a systematic screen of potential chemical modulators using GDSC data. This dataset contains gene expression profiles and IC_50_ information corresponding to 139 drugs for 707 different cell lines. As a proof of concept, we first calculated a DRS_wortmannin_ (MCF7) for each of the 39 breast-derived cell lines available in the dataset, and correlated DRS_wortmannin_ with Akt Inhibitor VIII IC_50_ across these cell lines. We found that DRS_wortmannin_ was significantly anti-correlated with the IC_50_ of Akt inhibitor VIII (PCC = –0.47, *P* = 2E-3) (Figure 6A). This indicates that increased DRS_wortmannin_ (lower PI3K-Akt-mTOR pathway activity) is associated with increased sensitivity to Akt inhibitor VIII. From this result we speculate that dual inhibition of PI3K and Akt may be more effective in decreasing the rate of cell proliferation. Potentially, Akt can also be phosphorylated in a PI3K-independent fashion suggesting that Akt activity may not be completely shut off by inhibiting PI3K [34]. Thus, inhibiting both proteins may result in the blockade of multiple downstream pathways that also play a role in cell proliferation, evasion of apoptosis, and metastasis.

To extend this analysis to all drugs available in the GDSC dataset, we systematically calculated a DRS for all cell lines and correlated the DRS_wortmannin_ with the IC_50_ of all 139 GDSC drugs [35]. In total, we found 16 drugs to be significantly correlated with DRS_wortmannin_ at *P* < 0.05, such that as DRS_wortmannin_ of the cell lines increased, the IC_50_ of the drug also increased (Figure 6B). We also found 37 drugs to be significantly anti-correlated with DRS_wortmannin_ at *P* < 0.05, suggesting that high DRS_wortmannin_ cell lines were more sensitive to these drugs (Figure 6B). To note, we observed that the IC_50_ profiles of lapatinib, a dual EGFR/HER2 inhibitor, and erlotinib, an EGFR inhibitor, were among the most anti-correlated with DRS_wortmannin_ [36, 37]. Furthermore, these drugs are FDA approved for use in the treatment of various cancers. This suggests that targeting receptor tyrosine kinases upstream of PI3K-signaling pathways may synergize with PI3K inhibition. Indeed, studies have shown that PI3K inactivation results in HER2 overexpression, or vice versa, and dual inhibition of HER2 and PI3K yields more optimal anticancer activity in breast cancer cell lines [38–40].

Furthermore, we postulated that groups of drugs with shared pharmacological characteristics would be correlated with DRS_wortmannin_ in a similar manner. Indeed, we found that chemotherapy drugs tend to be positively correlated with DRS (Figure 6B). This indicates that pharmacologically similar drugs could be grouped together based on their association with DRS. As such, this introduces new avenues for drug repositioning whereby, new potential drug candidates can be identified via correlation of their IC_50_ with DRS.

## DISCUSSION

In this study, we describe an analysis framework that utilizes protein inhibition gene expression profiles to probe the activity of the PI3K-Akt-mTOR pathway in primary breast tumors. Several studies have utilized perturbation gene expression profiles derived from primary tissue or cell lines that contain mutations in a gene of interest to probe activity of a pathway involving that gene in patient tumors [12, 41]. Furthermore, perturbation profiles derived from RNAi experiments have also been applied to study pathway misregulation in cancer [42–44]. However, there has been no systematic analysis that has utilized protein inhibition profiles created through treatment with small molecule inhibitors as an approach to measure cancer-associated pathway activity in primary tumors.

We argue that the use of protein inhibition gene expression profiles is more informative compared to mutation-based gene expression profiles in that perturbation of the protein of interest is independent of other molecular events. In our analysis, we showed that PTEN expression can confound the use of *PIK3CA* mutation as a marker for PI3K-Akt-mTOR pathway activity. This suggests that there may be additional mutations or molecular events that accompany, or may even cause, the mutation of interest. Indeed, we did not find any significant difference in pAkt-Akt ratio between *PIK3CA* mutant and wild type samples in both ER+ and ER- tumors, as we did when stratifying on DRS (Supplementary Figure S2). Furthermore, protein inhibition perturbation profiles encode information different from that encoded in RNAi-based perturbation profiles. The use of RNAi completely ablates protein production resulting in gene expression profiles that also capture information about the disruption of protein-protein interactions and other non-catalytic functions, which may affect the overall transcriptomic signature and complicate downstream analyses. Therefore, small molecular inhibitors may inhibit the catalytic activity of a protein without interfering with its potential regulatory functions, whereas this is not possible with RNAi [14]. Hence, we claim that protein inhibition profiles captures only the downstream effects of protein catalytic activity disruption, and not the effects caused by interfering with protein-protein interactions. However, this postulation remains to be directly tested in follow-up studies that utilize RNAi treatment gene expression profiles.

Indeed, there exist several issues associated with the use of small molecule inhibitors. First, there is the possibility of off-target effects, which add noise to the final drug treatment profile. Second, treatment with varying concentrations of the chemical inhibitor can result in differences in final gene expression output. Third, the three inhibitors all have different mechanisms by which they inhibit the PI3K-Akt-mTOR pathway. Wortmannin is a non-reversible inhibitor of PI3K, LY-294002 is a reversible inhibitor of PI3K, and sirolimus targets mTOR further downstream of PI3K. Thus, the gene expression output induced by pathway inhibition will differ due to variation in mechanisms of drug action. As such, we applied all three drug treatment profiles in our analysis to achieve greater sensitivity. We reasoned that using multiple drug treatment profiles to generate three DRSs for each patient will enable greater sensitivity in detecting significant differences in PI3K pathway activity between different tumor subgroups. Thus, we use the most significant DRS profile to estimate PI3K activity in tumor samples.

In spite of these limitations, using drug treatment profiles to probe activity of cancer-associated pathways provides an opportunity to study the transcriptomic effects of protein inhibition in the context of cancer. Our analysis framework can be extended to study other pathways in different cancers given that the appropriate drug treatment profiles exist. On this note, we also used drug treatment profiles from PC3 cell lines (available in CMap data) to calculate patient DRS in a prostate cancer dataset published by Taylor et al. [45]. We found that patient DRS_wortmannin_ and DRS_sirolimus_ was significantly anti-correlated with their Gleason scores indicating that increased PI3K activity was associated with disease severity (Supplementary Figure S3). Furthermore, we utilized drug treatment profiles from HL60 cell lines to calculate patient DRS and predict prognosis in several acute myeloid leukemia datasets. However, the survival results were not consistent across datasets perhaps due to the fact that the most differentially expressed genes in the HL60 drug treatment profile were not enriched for genes involved in the PI3K-Akt-mTOR pathway. A possible reason may be that off-target effects were more pronounced in HL60 cell lines leading to low quality drug treatment profiles.

In summary, we performed a systematic proof-of-concept study showing that drug treatment profiles are valuable tools that can provide insight into breast cancer pathway dysregulation. Our approach is novel in that we utilize protein inhibition profiles that provide different information that may not be present in RNAi- or mutation-based activity profiles. We validate the predictiveness of the DRS metric using RPPA data and show that DRS can predict patient prognosis, response to chemotherapy, and provide insight into the action of other potential anti-cancer drugs.

## MATERIALS AND METHODS

### Data acquisition

Raw CMap drug treatment profiles (.CEL files) for MCF7, HL60, and PC3 cell lines were downloaded from the CMap data portal (https//www.broadinstitute.org/cmap) [32]. Only drug treatment profiles that corresponded to the highest treatment concentration were used, with the reasoning that any changes in gene expression will saturate at higher concentrations. Release 5.0 of the GDSC gene expression and IC_50_ data was downloaded from http://www.cancerrxgene.org/downloads/ [35]. The normalized METABRIC gene expression dataset (*n* = 2136) was downloaded from the European Genome-Phenome Archive (http://www.ebi.ac.uk/ega) under the accession number EGAS00000000083 [15]. Level 3 TCGA breast cancer gene expression (RNA-seq, *n* = 1037)) and RPPA data (*n* = 408) were downloaded from the TCGA data portal (https://tcga-data.nci.nih.gov/tcga/) [18]. Normalized gene expression data by van de Vijver et al. was downloaded from the Netherlands Cancer Institute's data portal (*n* = 260) (http://ccb.nki.nl/data) [19]. Normalized gene expression data from primary breast tumors published by Loi et al. was downloaded from GEO under accession number GSE6532 (*n* = 255) [17]. Gene expression data and information about residual cancer burden used to evaluate DRS as a predictive marker was published by Hatzis et al. and downloaded from GEO under the accession numbers GSE25065 and GSE25066 (*n* = 508) [16]. Prostate cancer data by Taylor et al. was downloaded from GEO under accession number GSE21032 (*n* = 179) [45]. All gene expression datasets contained time-to-event survival and clinicopathological information on cancer patients.

### Pre-processing and generation of drug treatment profiles

Drug treatment gene expression profiles (.CEL) corresponding to wortmannin, LY-294002, and sirolimus from CMap were background corrected using Robust Microarray Analysis (RMA) and quantile normalized. Each probe set was fitted with a multichip linear model and collapsed based on mean probe set intensity. All pre-processing steps were implemented in the R programming environment using the Bioconductor package “affy” [46]. After normalization, drug treatment profiles were generated by: (1) Taking the log_2_ ratio of treatment vs. control for all genes (2) Labelling values > 0 as the “up-regulated” group and values < 0 as the “down-regulated” group (3) z-transforming ratios into z-scores to derive *p*-values for each gene (4) Implementing –log_10_ transformation to *p*-values, and (5) Trimming transformed *p*-values to so that they fall within [–20, 20] to generate a final gene expression profile. All replicate drug treatment profiles were averaged and drug treatment profiles corresponding to the highest treatment concentration were used in subsequent analyses. RPPA data was log_10_-transformed before analysis.

### Calculation of tumor sample DRS

A DRS is calculated for each drug treatment profile-tumor sample pair based on the IDEA framework [47]. Given the sorted tumor gene expression profile of a single patient sample g = [g_1_, g_2_, g_3_…g_i_…g_n_] and a drug treatment profile d = [d_1_, d_2_, d_3_…d_i_…d_n_] (sorted according to g), we generate a foreground function f(i) and a background function b(i) for the up- and for the down-regulated group:

$$f\left(i\right)=\frac{\sum_{j=1}^{i}\left|g_{j}d_{j}\right|}{\sum_{j=1}^{n}\left|g_{j}d_{j}\right|},1\leq i\leq n$$ (1)

$$b\left(i\right)=\frac{\sum_{j=1}^{i}\left|g_{j}\left(1-d_{j}\right)\right|}{\sum_{j=1}^{n}\left|g_{j}\left(1-d_{j}\right)\right|},1\leq i\leq n$$ (2)

The pre-DRS (pDRS) is then calculated using the following equations:

$$pDRS_{up/dn}^{+}=\text{max}\left[f\left(i_{\text{max}}\right)-b\left(i_{\text{max}}\right),0\right],\text{ where }i_{\text{max}}=\text{arg }{\text{max}}_{i=1,2,3\ldots n}\left[f\left(i\right)-b\left(i\right)\right]$$ (3A)

$$pDRS_{up/dn}^{-}=\text{min}\left[f\left(i\text{min}\right)-b\left(i\text{min}\right),0\right],\text{ where }i_{\text{min}}=\text{arg }{\text{min}}_{i=1,2,3\ldots n}\left[f\left(i\right)-b\left(i\right)\right]$$ (3B)

$$pDRS_{up/dn}=\{\begin{array}{l} pDRS^{+},pDRS^{+}>\left|pDRS^{-}\right|\\ pDRS^{-},otherwise \end{array}$$ (3C)

We then normalize the pDRSs (of both the up- and down-regulated profiles) first by permuting the genes in the tumor expression profile 1000 times to yield a null pDRS distribution. The pDRSs were then divided by the null pDRSs to yield a DRS_up_ and a DRS_dn_ profile. The difference between DRS_up_ and DRS_dn_ was computed to yield the final DRS. Each patient sample was assigned three DRSs, each corresponding to one of the three PI3K-Akt-mTOR pathway inhibitors.

### Association of DRS with survival and clinical phenotypes

Survival analysis was implemented by fitting Kaplan-Meier estimators to patient survival information. Patients were stratified at DRS = 0 and significant differences in survival were evaluated using the log-rank test. For the tissue-specific survival analysis, univariate Cox proportional hazards models were fitted to patient DRS to predict patient survival and significance was calculated using the Wald test. *P*-values were adjusted for multiple hypothesis testing using the Benjamini-Hochberg procedure. Survival analysis was conducted using the “survival” R package. Survival data in GSE21032 was limited so DRS was correlated with the Gleason score of the prostate tumors instead, using spearman correlation across 150 patient samples [45].

### Machine learning analysis

Machine learning analysis was performed in the Hatzis dataset using MCF7/LY-294002 DRS as features to classify patients into those with high residual cancer burden (RCB-II/III) and low residual cancer burden (RCB-0/I) [16]. The random forest learning method was used to classify patients into the two cancer burden groups. 10-fold cross validation was used to calculate the AUC. The random forest algorithm was implemented using the R package “randomForest”.

### Correlation analysis and clustering of GDSC drugs

The GDSC dataset contained gene expression data and drug IC_50_ information for 648 cell lines and 139 drugs, respectively [35]. The wortmannin drug treatment profiles from CMap were used to calculate a DRS for each cell line [32]. Correlation of DRS with Akt Inhibitor VIII was calculated using Pearson correlation across 39 breast-derived cell lines. Correlation of DRS with IC_50_ of all GDSC drugs in the drug screen was calculated using Spearman correlation across all 648 cell lines regardless of tissue type. Potential PI3K inhibitor modulators were selected using an adjusted *P* < 0.05 cutoff (Benjamini-Hochberg corrected) from the correlation analysis.

## SUPPLEMENTARY MATERIALS FIGURES AND TABLES


