# Supplementary material for: Application of pharmacologically induced transcriptomic profiles to interrogate PI3K-Akt-mTOR pathway activity associated with cancer patient prognosis
Source: Oncotarget. 2016 Aug 31;7(51):84142–54. doi: 10.18632/oncotarget.11776 (PMC5356650; doi:10.18632/oncotarget.11776)
Supplement: Supplementary file 1 [file oncotarget-07-84142-s001.pdf]

Application of pharmacologically induced transcriptomic profiles to interrogate PI3K-Akt-mTOR pathway activity associated with cancer patient prognosis

Supplementary Materials

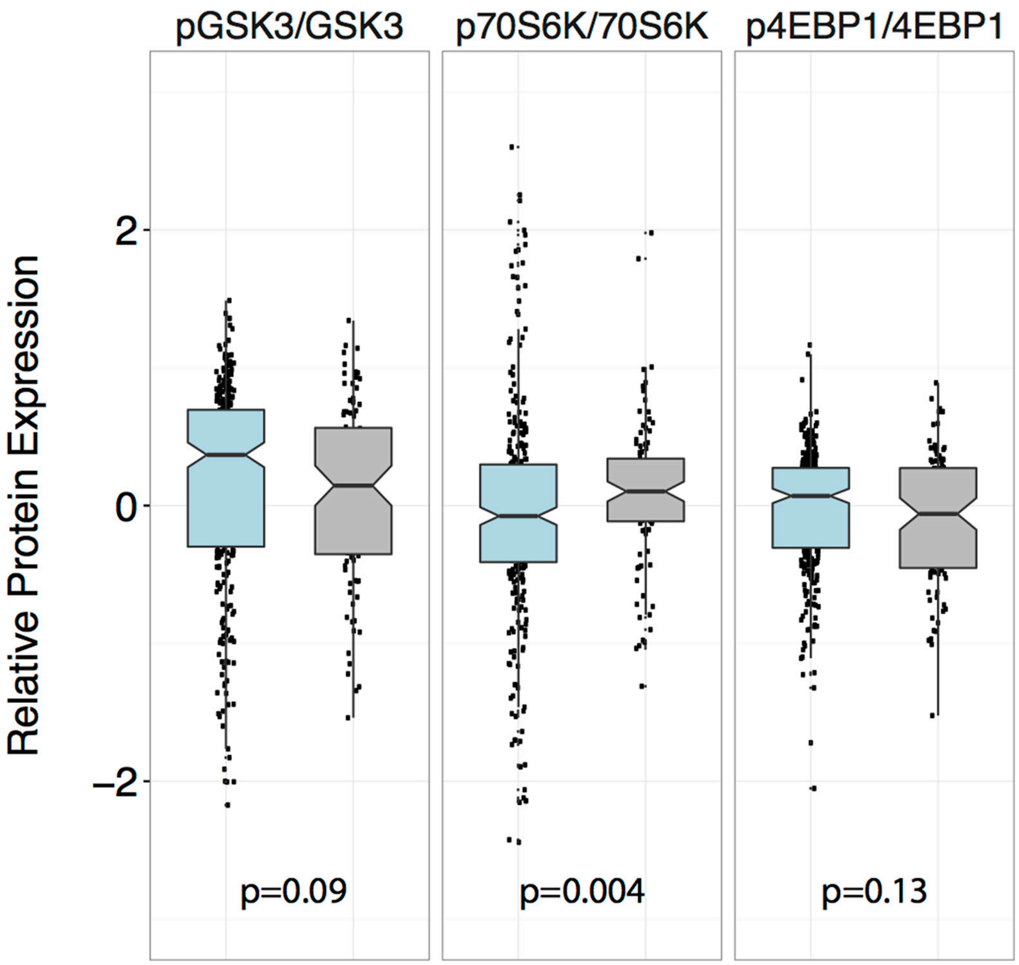

Supplementary Figure S1: Boxplot comparison of pGSK, p70S6K, and p4EBP1 protein expression ratios between ER+ and ER- breast cancer samples.

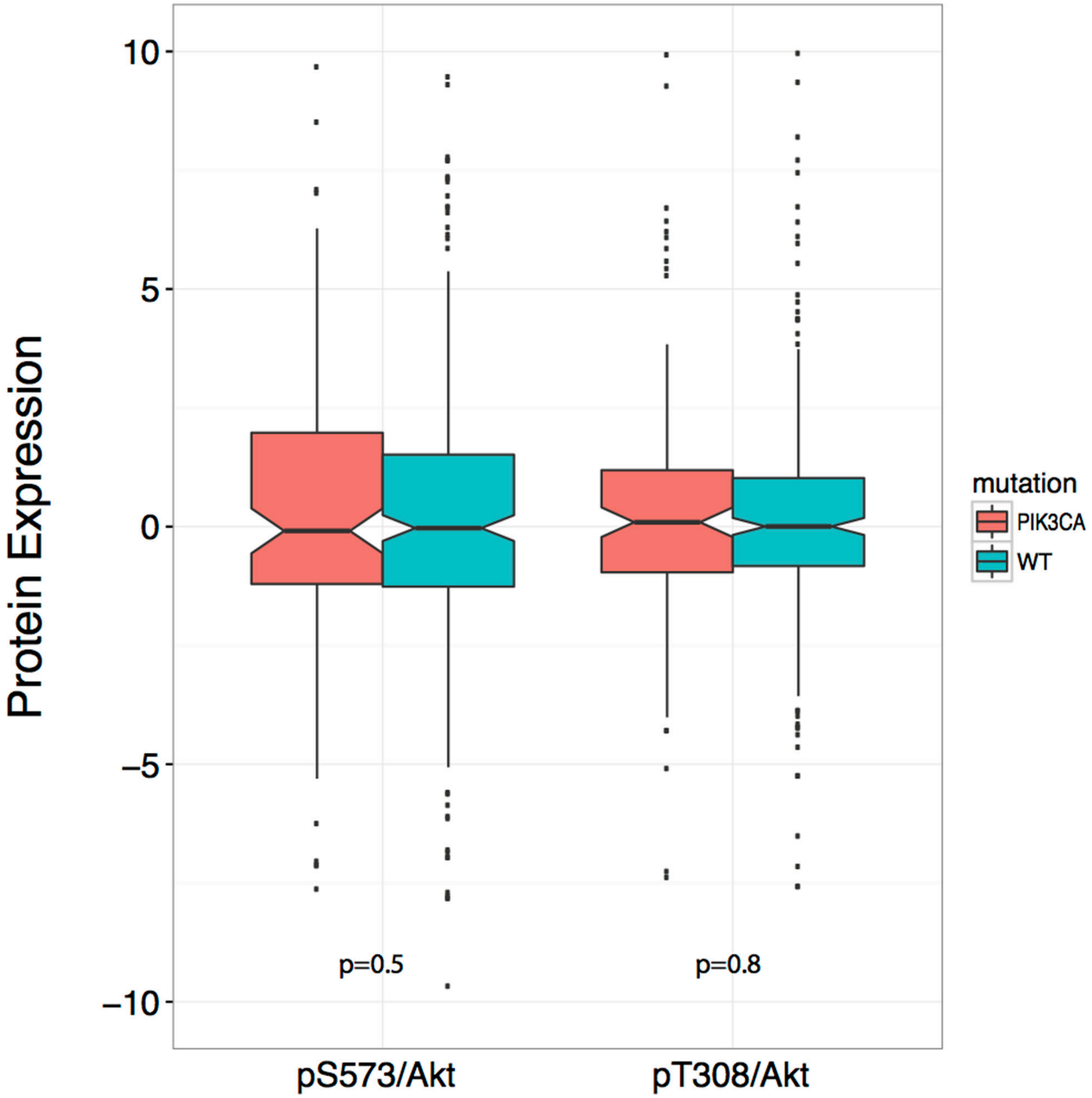

Supplementary Figure S2: Boxplot comparison of pAkt (S573 and T308) protein expression ratios between *PIK3CA* mutant and wild-type breast cancer tumor samples.

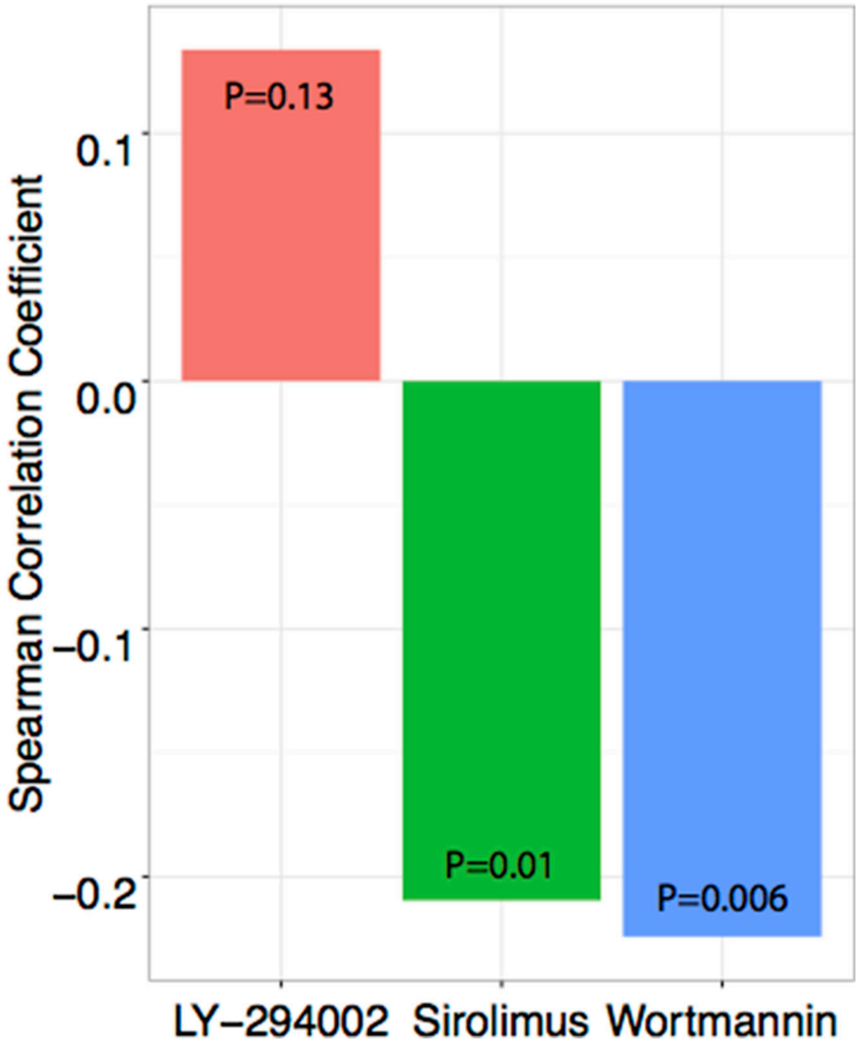

Supplementary Figure S3: Correlation of PI3K-Akt-mTOR pathway inhibitor DRS with Gleason scores in primary prostate tumors. Barplot of Spearman correlation coefficients between patient Gleason score and LY-294002, Sirolimus, and Wortmannin DRS, respectively.

Supplementary Table S1: Univariate Cox regression analysis in breast cancer patients (Curtis) using DRS generated from nine different drug/cell-line combinations

Drug	Univariate Cox Regression Analysis		
	**Cell Line**	**HR (95% CI)**	***P* Value**
	MCF7	0.96 (0.95–0.97)	6.32E-21
Wortmannin	PC3	0.96 (0.95–0.97)	6.30E-12
	HL60	0.98 (0.96–0.99)	2.90E-04
	MCF7	0.97 (0.95–0.99)	5.60E-04
LY-294002	PC3	1.03 (1.01–1.05)	2.90E-03
	HL60	0.99 (0.96–1.02)	5.30E-01
	MCF7	0.95 (0.94–0.96)	2.03E-16
Sirolimus	PC3	0.97 (0.96–0.98)	2.00E-12
	HL60	0.95 (0.94–0.97)	1.10E-08

Abbreviations: HR = hazard ratio; 95% CI = 95% confidence interval.
